# A short screening tool identifying systemic barriers to distress screening in cancer care

**DOI:** 10.1002/cam4.6331

**Published:** 2023-07-12

**Authors:** Felice Simnacher, Anna Götz, Sabine Kling, Jan Ben Schulze, Roland von Känel, Sebastian Euler, Moritz Philipp Günther

**Affiliations:** ^1^ Department of Consultation‐Liaison Psychiatry and Psychosomatic Medicine, University Hospital Zurich University of Zurich Zurich Switzerland; ^2^ Computer Vision Laboratory, Department of Information Technology and Electrical Engineering Swiss Federal Institute of Technology (ETH) Zurich Zurich Switzerland

**Keywords:** cancer, distress screening, hospital administrators, principal component analysis, psycho‐oncology

## Abstract

**Introduction:**

International guidelines on cancer treatment recommend screening for early detection and treatment of distress. However, screening rates are insufficient. In the present study, a survey was developed to assess perceived systemic barriers to distress screening.

**Methods:**

A three‐step approach was used for the study. Based on qualitative content analysis of interviews and an expert panel, an initial survey with 53 questions on barriers to screening was designed. It was completed by 98 nurses in a large comprehensive cancer center in Switzerland. From this, a short version of the survey with 24 questions was derived using exploratory principal component analysis. This survey was completed by 150 nurses in four cancer centers in Switzerland. A confirmatory factor analysis was then performed on the shortened version, yielding a final set of 14 questions.

**Results:**

The initial set of 53 questions was reduced to a set of 14 validated questions retaining 53% of the original variance. These 14 questions allow for an assessment within 2–3 min that identifies relevant barriers to distress screening from the perspective of those responsible for implementation of distress screening. Across several hospitals in Switzerland, the timing of the first distress screening, lack of capacity, patient and staff overload, and refusal of distressed patients to be referred to support services emerged as major problems.

**Conclusion:**

The validated 14 questions on barriers to screening cancer patients for distress enable clinicians and hospital administrators to quickly identify relevant issues and take action to improve screening programs.

## Graphical abstract

In this study we developed a new ultra short survey (14 questions) to identify (and possibly resolve) barriers to screening for distress in patients with cancer. Despite international guidelines recommending screening for distress in all cancer patients, screening rates are low internationally. The survey presented should be a tool for hospital administrators to rapidly identify reasons for low screening rates and implement remedies. The study also presents results from surveying a total of 248 nurses in Switzerland responsible for distress screening on their perceived barriers. Across several hospitals in Switzerland, the timing of the first distress screening, lack of capacity, patient and staff overload, and refusal of distressed patients to be referred to support services emerged as major problems.

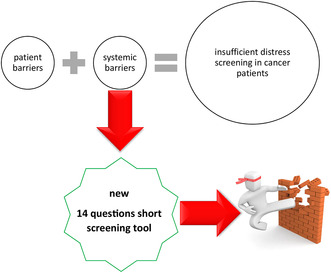

## 1 INTRODUCTION

Psychological distress in cancer is defined as a broad spectrum of unpleasant experiences of psychological, social, physical, or spiritual nature including depression, anxiety, or other mood adjustment disorders.^1^ About half of patients with cancer show a significant level of distress.^1^, ^2^, ^3^ Distress often remains unrecognized in primarily somatic treatment settings, and frequently, there is an unmet need for psychosocial support.^4^, ^5^, ^6^, ^7^ Earlier referral to psychosocial care is associated with better outcomes such as less anxiety and greater health‐related quality of life.^1^

Therefore, consensus‐based treatment guidelines have recommended distress screening to recognize psycho‐oncological needs as part of standard cancer treatment.^7^, ^8^, ^9^ Consequently, screening for distress is part of the requirement for accreditation of cancer treatment centers. For screening, the distress thermometer (DT) has been developed by the National Comprehensive Cancer Network.^10^ The problem list (PL) usually accompanies the DT and consists of five different categories of problems (namely practical, family, emotional, spiritual/religious, and physical problems) to help identify patients' source of distress.^10^ DT and PL are internationally used standardized screening tools, allowing for a quick assessment for distress.^1^, ^11^

Yet, research has shown screening rates to range between 22% and 74% in outpatient settings and 40% in inpatient settings.^12^ Reasons include patient‐related factors, such as cognitive abilities, psychological factors, gender, age, individual preferences, physical or language barriers.^12^, ^13^ On the contrary, there are systemic barriers to screening, specific to individual treatment centers, often including inconsistencies in the implementation of screening programs and inadequate timing of screening.^14^ The present study focuses on such systemic barriers. It aims to provide an instrument to identify such systemic issues efficiently in any one treatment center.

In many cancer treatment centers, cancer care nursing staff and radiographers are responsible for distress screening,^15^ but it could also be done cancer physicians. It is rarely done by psychotherapists (psychiatrists, psychologists, social‐workers), as screening is done to distinguish between patients needing attention from the latter and those not requiring such psycho‐oncological support. In order to optimize the screening process, it is crucial to identify barriers to screening from the perspective of those responsible for its practical implementation. As such barriers, Australian nurses named insufficient human resources (38%), time (24%), training (19%), evidence of usefulness (5%), and documentation (4%).^16^ Similar results were reported in qualitative research.^17^, ^18^ In a study from Japan, care teams of 422 hospitals criticized insufficient resources to address problems identified via screening (66%), patients having difficulties verbalizing sources of distress (58%), insufficient time (49%), patients’ refusal of referrals (40%), problems with implementation of screening strategies (54%), insufficient human resources (45%), and insufficient knowledge (29%).^19^ In a study with 72 interviews with oncologists and nurses, the following challenges were identified: too much paperwork, patient opposition, cultural and language barriers, technological barriers, insufficient time, and lack of automatic referral.^20^

The primary objective of this study was to develop a questionnaire to identify barriers in guideline‐based distress screening observed by those responsible for screening within 2–3 min. While there is abundant research on patient‐related barriers to screening, and some on systemic barriers to screening, there is no instrument (to the best of our knowledge) to identify treatment center specific systemic barriers. Prior to the present study, a series of interviews with nine nurses and four nurse experts was conducted at a large comprehensive cancer center in Switzerland and results published as a qualitative pre‐study.^21^ From these qualitative findings, an expert consensus group (composed of nurses, psychologists, and physicians in cancer care and psycho‐oncology) derived 53 relevant questions on systemic barriers. A three‐step approach was then used to derive a validated short survey instrument from the initial set of 53 questions.

A secondary objective of the present study was to provide new data on barriers nurses experience in screening for distress in four hospitals in Switzerland.

## 2 MATERIALS AND METHODS

### 2.1 Study procedure and sources of data

**FIGURE 1:**
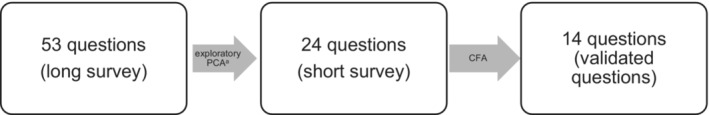
Flowchart of statistical analysis. Statistical analysis is described in the methods section. ^a^PCA, principal component analysis; CFA, confirmatory factor analysis.

The study was conducted in three steps (see Figure 1).

In a first step, 53 questions originating from a qualitative pre‐study,^21^ augmented by a group of experts (e.g., nurses, psychologists, and physicians) working with cancer patients at the Comprehensive Cancer Center Zürich (CCCZ), were answered by 98 nurses working at the CCCZ between March and June 2021. The original online survey was in German, and the translated English version is provided in the Table S1. The CCCZ includes 17 accredited organ centers serving in‐ and outpatients. The CCCZ integrates research activities and treatments for more than 15,000 patients with cancer annually. The CCCZ is certified according to the guidelines of the German Cancer Society.^22^

In a second step, the 53‐item survey was trimmed to 24 items (19 statements on barriers in screening, 5 on demographic characteristics; see Table S2) using principal component analysis (PCA; see statistics). This 24‐item short version was completed by a new group of 150 participating nurses from four large, certified cancer treatment centers in German speaking parts of Switzerland between October 2021 and January 2022.

All participating nurses consented to complete both the long and short version of the survey questionnaire voluntarily and anonymously. The Cantonal Ethics committee of Zurich reviewed the project and ascertained that it falls outside the scope of the current laws on human research (BASEC Nr. Req‐2020‐01228). At all four treatment centers, the DT and PL are used for screening at the day of admission, every second week thereafter, and in case of significant events during treatment (e.g., new diagnosis, unfavorable treatment results, and progression of disease). Whenever distress in the DT is 5 or more, referral to spiritual care, psycho‐oncological services, or social services will be initiated based on the source of distress identified via the PL. Patients with moderate to severe levels of distress (cutoff value ≥5) are re‐screened after 7 days.

In a third and final step, a confirmatory factor analysis (CFA) of the short 24‐item version was conducted, providing a final 14‐item survey.

### 2.2 Data preparation

For data preparation, age and years of experience in cancer care (but not necessarily distress screening) were converted from continuous variables into five‐level categorical items with bins widths of approximately equal numbers of observations. Outliers with three standard deviations above/below ample mean were removed and missing data of less than 30% were imputed using multiple imputations by chained equations (MICE),^23^ considered to be one of the best approaches to deal with missing data.^24^

### 2.3 Statistical analysis

Exploratory PCA was conducted using the FactoMineR package^25^ in RStudio (version 1.4.1717) to identify the most statistically significant questions from a set of 53 questions (see Table S1) answered by 98 participants. PCA is a method of multivariate analysis to perform a linear transformation of variables to structure and simplify a data set.^26^ The goal is to reduce the total number of variables (here questions) while keeping as much of the variation present in the data set as possible.^26^ The reduction is realized by linear combinations of the original questions which then result in new variables, the so‐called principal components.^26^ The extracted model is considered a good fit, if the removal of one principal component leads to a substantial deterioration and the addition of an additional principal component to only a marginal improvement. In the present study, this was the case with six principal components, using an eigenvalue of two or more,^27^ as can be seen in the scree plot in the Figure S1. The six principal components identified (see Table S7) related to doubts in the clinical benefits of screening, doubts in the screening procedure, socio‐economic factors of screeners, issues in the relationship between patients and those responsible for screening, an apparent lack of training of screeners and difficulties in dealing with emotions of patients during screening. These principal components also cover the conceptual categories of the initial 53‐item survey (sociodemographic data, screening in general, screening procedure, discussion of screening results with patients, referral of distressed patients, and the effect of screening results on everyday patient care). Subsequently, for each of the extracted principal components, two to four questions were selected, resulting in the 24‐item intermediate survey. The selected questions had to have a relatively high loading based on the data set obtained on the long version of the survey (Table S7) and represent the principal component well.

For validation of these extracted principal components, CFA was performed on the data of the 24‐item version of the survey, resulting in 14 validated questions (Table S2). This analysis was conducted in RStudio using the lavaan package.^28^

CFA removes questions showing non‐significant loadings based on the data set obtained on the 24‐item version of the survey in iterative steps, until a good model fit is obtained. This resulted in 14 confirmed questions. In the final set of these 14 questions, chi‐squared test with a test statistic of 105(62) < 0.001 and RMSEA of 0.08 suggested a good overall model fit. Similarly, the comparative fit index (CFI) of 0.92 fulfills the criteria for good model fit (0.90–0.95). Summary statistics and resulting principal component loadings are presented in the Table S8. Overall, using exploratory PCA and CFA, it was thus possible to reduce the number of questions to 14, while retaining 53% of the original variance.

In addition (and independent of PCA), barriers encountered by nurses to screening patients for distress were explored via descriptive statistics in SPSS (version 26).

## 3 RESULTS

### 3.1 Demographic characteristics

The long version of the survey was completed by 98 nurses from the CCCZ. The demographic characteristics are reported in Table S3. The short version of the survey was completed by 150 participants. See Table S4 for demographic characteristics.

### 3.2 Descriptive statistics

**FIGURE 2:**
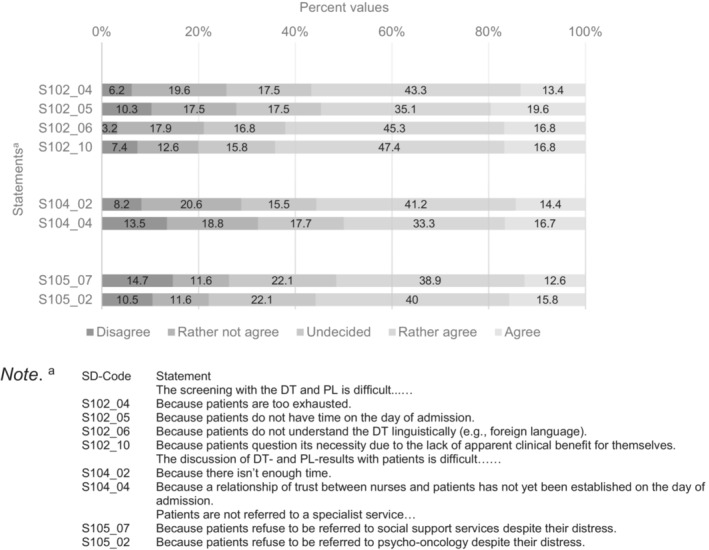
Statements of the long version of the survey 50% of the participants “rather agree” or “agree” with.

Figure 2 shows statements 50% of respondents to the initial 53‐item survey indicated they “rather agree” or “agree” with and less than 30% of participants disagreed or tended to disagree with. Table S5 provides distribution of responses from all participants answering the initial 53‐item version of the survey; Table S6 provides the distribution of responses from all participants answering the 24‐item version of the survey.

It seems that statements receiving high agreement in the long version were also included in the short version of the survey, where they found similarly high agreement (see Table S6, marked in bold).

**FIGURE 3:**
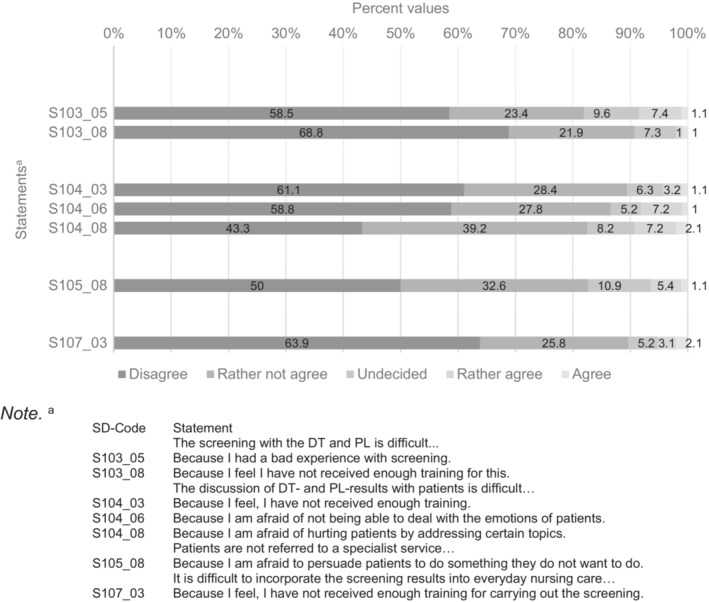
Statements of the long version of the survey over 80% of the participants “disagree” or “rather not agree” with.

Statements in Figure 3 were rated with “disagree” or “rather not agree” by over 80% of participating nurses and less than 10% “rather agreed” or “agreed,” again with data drawn from respondents to the initial 53‐item survey. Thus, nurses indicated these topics were not the main problems encountered in distress screening.

### 3.3 Final version of the survey

The validated 14‐item survey is presented below in Table 1.

**TABLE 1:** Validated 14‐item version of the survey.

Question/Statement	Code	Possible responses
1. How old are you?		
	SD02_01	I am … years
2. What is your professional qualification? Select the highest educational qualification you have achieved so far		
	SD12_01	Student
	SD12_02	Nurse with HF/DN II
	SD12_03	Nurse with bachelor's degree
	SD12_05	Nurse with master's degree
	SD12_06	Nurse with MAS/HöFa
	SD12_07	Different
3. How many years of professional experience do you have?		
	SD20_02	I have been working in nursing for… years
Screening with the DT and PL is difficult…		
4. Because screening is repeated too frequently	S102_13	Disagree, rather not agree, undecided, rather agree, strongly agree
5. Because the burden for relatives is not inquired about	S102_11	Disagree, rather not agree, undecided, rather agree, strongly agree
Screening with the DT and PL is difficult…		Disagree, rather not agree, undecided, rather agree, strongly agree
6. Because I question its necessity due to the lack of apparent clinical benefit	S103_01	Disagree, rather not agree, undecided, rather agree, strongly agree
7. Because screening means additional work for me without any additional benefit	S103_04	Disagree, rather not agree, undecided, rather agree, strongly agree
The discussion of DT‐ and PL‐results with patients is difficult…		
8. Because there is no private location for conversations	S104_01	Disagree, rather not agree, undecided, rather agree, strongly agree
9. Because a relationship of trust between nurses and patients has not yet been established on the day of admission	S104_04	Disagree, rather not agree, undecided, rather agree, strongly agree
10. Because I feel uncomfortable talking about topics such as sexuality	S104_05	disagree, rather not agree, undecided, rather agree, strongly agree
11. Because I am afraid of not being able to deal with the emotions of patients	S104_06	Disagree, rather not agree, undecided, rather agree, strongly agree
Patients are not referred to a specialist service…		
12. Because it is not entirely clear what the specialized services can offer	S105_01	Disagree, rather not agree, undecided, rather agree, strongly agree
It is difficult to incorporate the screening results into everyday nursing care…		Disagree, rather not agree, undecided, rather agree, strongly agree
13. Because I lack options for action	S107_04	Disagree, rather not agree, undecided, rather agree, strongly agree
14. Because there is insufficient interdisciplinary collaboration on psychosocial problems	S107_05	Disagree, rather not agree, undecided, rather agree, strongly agree

## 4 DISCUSSION

In this study, an initial set of 53 questions on common barriers to screening cancer patients for distress was derived from prior qualitative research^21^ and subsequently reduced to a set of 14 validated questions which can be answered in 2–3 min. Both surveys help identify cancer treatment center specific systemic barriers to screening for distress. However, the short version may spare time and resources, allowing optimizers to interview all employees involved in screening, even in very large treatment centers.

The time needed to answer an individual question ranged from 7 (long version) to 13 s (short version). The observation that participants invested more time in answering the short version concurs with the literature. It has been shown that participants in shorter surveys generally take more time to answer each question compared to participants in longer surveys.^29^ Potential reasons include increased motivation and deeper cognitive processing.^30^ This might signify nurses felt the short version was important for quality improvement and answered conscientiously.

With regard to systemic barriers to distress screening, 55% of nurses found there is too little time for both staff and patients on the day of admission to perform the screening and 50% found a trustful relationship has not yet been established on admission day. It may thus be more suitable for the first screening to be administered from the second day of admission, as has been suggested in prior research.^14^ However, early recognition of distress is essential for timely treatment, reported to have a positive effect on therapy adherence and physician‐patient‐communication.^1^ This trade‐off between requirements needs to be weighted in routine clinical practice.

In addition, 56% of nurses reported patients to often be too exhausted to complete the DT. This confirms prior research indicating poor screening rates in patients with psychiatric comorbidity who may feel fatigued more easily.^7^, ^31^ This means the patient's condition during the day and other planned procedures must be taken into account, when choosing a time of day for screening. Furthermore, translated versions of the DT and PL should be used whenever necessary, in order to eliminate language as a barrier to screening (reported by 62% of nurses).

Another barrier to screening seems to be patients questioning the necessity of screening due to the lack of apparent clinical benefit for themselves, or patients refusing a referral to a specialist service despite distress. This is unfortunate, since earlier screening/referral can have a positive impact on health‐related outcomes, such as less anxiety and better emotional well‐being and therefore coping with cancer.^32^ Further, with patients refusing to be referred to appropriate supportive services, screening has no benefits.^33^ This highlights the importance of clear communication with the patient concerning the purpose and benefits of screening and supportive services. Only if nurses are trained and ultimately convinced by the clinical benefit of screening (and specialized support services), can they sufficiently convince patients for the values of distress screening as well.

Survey statements more than 80% of the participating nurses “disagreed” or “rather not agreed” with seem to be less relevant problems encountered in distress screening. Such statements included bad experiences, dealing with patients' emotions and addressing certain themes in distress screening. The great majority of the nurses felt that they had received enough training to perform screening and to incorporate it into everyday work. However, this seems to be in contradiction to nurses bemoaning patients' lack of understanding of the benefits of screening and appropriate referral. There may be a discrepancy between nurses' self‐assessment of the training they received and the communication skills they acquired. To substantiate this assumption, future research should include the perspectives of patients and communication skills trainers.^34^ Training seminars for those responsible for screening should be carefully designed and validated.

This study has several limitations. The fact that 50 participants chose not to disclose their workplace may be an indication that they feared consequences at the workplace when mentioning barriers to screening. As a result, we chose not to disclose the sites of the three other cancer treatment centers from which nurses were recruited to participate in this study. Also, we only included nurses working in German speaking parts of Switzerland, which may limit generalizability of results to other cultural and language settings. Similarly, we did not interview responsible physicians or patients on their perception of screening (or no screening), which would allow to contrast the perception of nurses with that of these groups. In prior research interviewing patients on why they refused psycho‐oncological support despite indicating distress during screening, 46% believed they should be able to manage emotions independently, 24% claimed to receive sufficient help from other sources, and 23% perceived their distress to be too minor.^35^ In addition, fear of stigmatization^3^ or avoidant coping strategies^36^ may prevent patients from participation in screening or admitting distress. One study indicated patients with a mental disorder or those using psychopharmacology were least likely to be screened for distress.^7^ Recent research has concluded that screening alone without thoroughly informing patients on the nature of distress and available services is ineffective.^11^ Similarly, screening alone will not alleviate distress.^37^ Yet, patients not being screened are most likely to not be referred to psycho‐oncology.^38^ Finally, when using the short version instead of the long version of the survey to assess barriers to screening, 47% of the variance may be lost according to CFA. However, the short version takes less time for participants to complete and less time for analysis of results.

## 5 CONCLUSION

In summary, the validated 14 question survey allows a quick assessment of relevant barriers to distress screening and subsequent optimization of screening programs. Even in very large treatment centers, it should allow administration to receive feedback on screening procedures from all employees at little cost. Based on our results, timing of the first screening during inpatient treatment (and during the day) and allotment of sufficient time for both patients and staff to build up trust and executing screening may be key to successful implementation of guidelines on distress screening. Also, training should not only be considered sufficient by those receiving it (nurses), but should be followed up by appropriate performance evaluations. Results indicated refusal of distressed patients to be referred to support services is a major problem limiting efficacy of screening programs, but further research is needed on how proper training of staff responsible for screening and discussion of screening results with patients may alleviate this problem. Further studies in other parts of Switzerland and foreign countries on this topic are needed for definite recommendations. In addition, future research needs to interview patients on their opinion on screening procedures and available support services, particularly including the voice of those patients using such services.

## AUTHOR CONTRIBUTIONS

**Felice Simnacher:** Data curation (equal); formal analysis (supporting); methodology (supporting); visualization (lead); writing – original draft (equal). **Anna Götz:** Conceptualization (supporting); data curation (lead); formal analysis (supporting); methodology (supporting); visualization (supporting); writing – original draft (equal). **Sabine Kling:** Formal analysis (supporting); methodology (lead); software (lead); validation (equal); visualization (supporting); writing – review and editing (supporting). **Jan Ben Schulze:** Supervision (supporting); validation (supporting); writing – review and editing (supporting). **Roland von Känel:** Project administration (equal); resources (lead); supervision (supporting); writing – review and editing (supporting). **Sebastian Euler:** Project administration (supporting); resources (equal); supervision (supporting); writing – review and editing (supporting). **Moritz Philipp Günther:** Conceptualization (lead); data curation (lead); formal analysis (supporting); investigation (lead); methodology (lead); project administration (lead); resources (supporting); software (supporting); supervision (lead); validation (lead); visualization (lead); writing – original draft (lead); writing – review and editing (lead).

## FUNDING INFORMATION

No funding was obtained for this study.

## CONFLICT OF INTEREST STATEMENT

The authors declare that they have no competing interests.

## ETHICS STATEMENT

The Cantonal Ethics committee of Zurich reviewed the project and ascertained that it falls outside the scope of the current laws on human research (BASEC Nr. Req‐2020‐01228). No data from patients were used. Consent was obtained from all survey participants. The authors assert that all procedures contributing to this work comply with the ethical standards of the relevant national and institutional committees on human experimentation and with the Helsinki Declaration of 1975, as revised in 2008.

## Supporting information


Data S1.

## Data Availability

The data that support the findings of this study are available on request from the corresponding author. The data are not publicly available due to privacy or ethical restrictions.
