# Supplementary material for: A short screening tool identifying systemic barriers to distress screening in cancer care
Source: Cancer Med. 2023 Jul 12;12(16):17313–21. doi: 10.1002/cam4.6331 (PMC10501250; doi:10.1002/cam4.6331)
Supplement: Supplementary file 1 — Data S1. [file CAM4-12-17313-s001.docx]

# Supplement/ Appendix

**Appendix - Table 1**

*Long version of the survey (53 questions)*

| Sociodemographic data | Code | Possible responses |
| --- | --- | --- |
| At which hospital do you work? | V201 |  |
|  | V201_01  V201_02  V201_05  V201_06 | CCCZ  Treatment Center A  Treatment Center B  Treatment Center C |
|  |  |  |
| What is your sex? | SD01 |  |
|  | SD01_01  SD01_02  SD01_03 | Male  Female  Diverse |
|  |  |  |
| How old are you? | SD02 |  |
|  | SD02_01 | I am … years. |
|  |  |  |
| What is your professional qualification? Select the highest educational qualification you have achieved so far: | SD12 |  |
|  | SD12_01  SD12_02  SD12_03  SD12_05  SD12_06  SD12_07 | Student  Nurse with HF/DN II  Nurse with bachelor's degree  Nurse with master's degree  Nurse with MAS/HöFa  Different |
|  |  |  |
| How many years of professional experience do you have? | SD20 |  |
|  | SD20_02 | I have been working in nursing for… years. |

| Topic | Code | Statement that has to be assessed:  Each statement must be assessed. It should be indicated whether participant disagrees, rather not agrees, is undecided, rather agrees or strongly agrees. |
| --- | --- | --- |
| Screening in general | S102 |  |
| Application of the distress thermometer (DT) and problem list (PL) |  | Screening with the DT and PL is difficult... |
|  | S102_13 | Because screening is repeated too frequently. |
|  | S102_04 | Because patients are too exhausted. |
|  | S102_02 | Because there is no time for it. |
|  | S102_03 | Because other topics and deadlines have priority over the DT. |
|  | S102_05 | Because patients do not have time on the day of admission. |
|  | S102_06 | Because patients do not understand the DT linguistically (e.g., foreign language). |
|  | S102_07 | Because patients do not understand the DT cognitively (e.g., in case of delirium). |
|  | S102_08 | Because patients have difficulties in assessing their distress. |
|  | S102_09 | Because patients are too poorly informed about the meaning of screening through written explanations. |
|  | S102_10 | Because patients question its necessity due to the lack of apparent clinical benefit for themselves. |
|  | S102_11 | Because the burden for relatives is not inquired about. |
|  | S102_12 | Because screening should be done by another / specialized professional (e.g., doctors, nursing expert). |
| Screening procedure | S103 |  |
| Application of DT and PL, nurses' perspective |  | Screening with the DT and PL is difficult... |
|  | S103_01 | Because I question its necessity due to the lack of apparent clinical benefit. |
|  | S103_02 | Because I cannot support the screening guidelines. |
|  | S103_03 | Because patients will not answer honestly. |
|  | S103_04 | Because screening means additional work for me without any additional benefit. |
|  | S103_05 | Because I had a bad experience with screening. |
|  | S103_06 | Because in my opinion the presence of relatives influences the answers. |
|  | S103_07 | Because certain patient groups must be treated with caution. |
|  | S103_08 | Because I feel I have not received enough training for this. |
|  | S103_10 | Because patients are not referred for psycho-oncological support after all. |
| Discussion of the results | S104 |  |
| Personal exchange with patients |  | The discussion of DT- and PL-results with patients is difficult… |
|  | S104_01 | Because there is no private location for conversations. |
|  | S104_02 | Because there isn’t enough time. |
|  | S104_03 | Because I feel, I have not received enough training |
|  | S104_04 | Because a relationship of trust between nurses and patients has not yet been established on the day of admission. |
|  | S104_05 | Because I feel uncomfortable talking about topics such as sexuality. |
|  | S104_06 | Because I am afraid of not being able to deal with the emotions of patients. |
|  | S104_07 | Because I cannot offer appropriate interventions to the patients. |
|  | S104_08 | Because I am afraid of hurting patients by addressing certain topics. |
|  | S104_09 | Because I am not on the same wavelength with patients. |
|  | S104_10 | Because patients prefer to talk to other professionals (e.g., oncologists / doctors). |
|  | S104_11 | Because patients indicate a different level of distress than I subjectively perceive as a nurse. |
|  | S104_12 | Because topics are not addressed honestly and openly by the patient. |
|  | S104_13 | Because I am afraid to persuade the patients to do something they do not want to do. |
| Referral | S105 |  |
| Referral to specialized services |  | Patients are not referred to a specialist service… |
|  | S105_01 | Because it is not entirely clear what the specialized services can offer. |
|  | S105_08 | Because I am afraid to persuade patients to do something they do not want to do. |
|  | S105_07 | Because patients refuse to be referred to social support services despite their distress. |
|  | S105_02 | Because patients refuse to be referred to psycho-oncology despite their distress. |
|  | S105_03 | Because the referral to psycho-oncology must be done by physicians. |
|  | S105_04 | Because referrals to specialized services seem too expensive for the clinic. |
|  | S105_06 | Because social support services do not have enough capacity to see all patients who need it. |
|  | S105_05 | Because psycho-oncologists do not have enough capacity to see all patients who need it. |
| Screening results | S107 |  |
| Inclusion of screening results in everyday care |  | It is difficult to incorporate the screening results into everyday nursing care… |
|  | S107_01 | Because screening results are difficult to find in a patient's file. |
|  | S107_02 | Because there is insufficient professional support for acute psychosocial problems. |
|  | S107_03 | Because I feel that I have not received enough training for carrying out the screening. |
|  | S107_06 | Because I feel, I have not received enough training to interpret screening results. |
|  | S107_04 | Because I lack options for action. |
|  | S107_05 | Because there is insufficient interdisciplinary collaboration on psychosocial problems. |

**Appendix - Table 2**

*Short version (24 questions) and final validated version (in bold type) of the survey*

| Sociodemographic data | Code | Possible responses |
| --- | --- | --- |
| At which hospital do you work? | V201 |  |
|  | V201_01  V201_02  V201_05  V201_06 | CCCZ  Treatment Center A  Treatment Center B  Treatment Center C |
|  |  |  |
| What is your sex? | SD01 |  |
|  | SD01_01  SD01_02  SD01_03 | Male  Female  Diverse |
|  |  |  |
| **How old are you?** | SD02 |  |
|  | **SD02_01** | **I am … years.** |
|  |  |  |
| **What is your professional qualification? Select the highest educational qualification you have achieved so far:** | **SD12** |  |
|  | **SD12_01**  **SD12_02**  **SD12_03**  **SD12_05**  **SD12_06**  **SD12_07** | **Student**  **Nurse with HF/DN II**  **Nurse with bachelor's degree**  **Nurse with master's degree**  **Nurse with MAS/HöFa**  **Different** |
|  |  |  |
| **How many years of professional experience do you have?** | **SD20** |  |
|  | **SD20_02** | **I have been working in nursing for… years.** |

| Topic | Code | Statement that has to be assessed:  Each statement must be assessed. It should be indicated whether participant disagrees, rather not agrees, is undecided, rather agrees or strongly agrees. |
| --- | --- | --- |
| **Screening in general** | S102 |  |
| **Application of the distress thermometer (DT) and problem list (PL)** |  | **Screening with the DT and PL is difficult...** |
|  | **S102_13** | **Because screening is repeated too frequently** |
|  | S102_07 | Because patients do not understand the DT cognitively (e.g., in case of delirium). |
|  | S102_09 | Because patients are too poorly informed about the meaning of screening through written explanations. |
|  | S102_10 | Because patients question its necessity due to the lack of apparent clinical benefit for themselves. |
|  | **S102_11** | **Because the burden for relatives is not inquired about.** |
|  | S102_12 | Because screening should be done by another / specialized professional (e.g., doctors, nursing expert). |
| **Screening procedure** | S103 |  |
| **Application of DT and PL, nurses' perspective** |  | **Screening with the DT and PL is difficult...** |
|  | **S103_01** | **Because I question its necessity due to the lack of apparent clinical benefit.** |
|  | **S103_04** | **Because screening means additional work for me without any additional benefit.** |
|  | S103_06 | Because in my opinion the presence of relatives influences the answers. |
| **Discussion of the results** | S104 |  |
| **Personal exchange with patients** |  | **The discussion of DT- and PL-results with patients is difficult…** |
|  | **S104_01** | **Because there is no private location for conversations.** |
|  | S104_02 | Because there isn’t enough time. |
|  | **S104_04** | **Because a relationship of trust between nurses and patients has not yet been established on the day of admission.** |
|  | **S104_05** | **Because I feel uncomfortable talking about topics such as sexuality.** |
|  | **S104_06** | **Because I am afraid of not being able to deal with the emotions of patients.** |
| **Referral** |  |  |
| **Referral to specialized services** | **S105** | **Patients are not referred to a specialist service…** |
|  | **S105_01** | **Because it is not entirely clear what the specialized services can offer.** |
|  | S105_05 | Because psycho-oncologists do not have enough capacity to see all patients who need it. |
| **Screening results** |  |  |
| **Inclusion of screening results in everyday care** |  | **It is difficult to incorporate the screening results into everyday nursing care…** |
|  | S107_01 | Because screening results are difficult to find in a patient's file. |
|  | **S107_04** | **Because I lack options for action.** |
|  | **S107_05** | **Because there is insufficient interdisciplinary collaboration on psychosocial problems.** |

Validated questions are in bold type.

**Appendix - Table 3**

*Demographic characteristics long version of survey (without imputation of missing values)*

| Demographic characteristic | No. of participants | Valid percent values^a^ |
| --- | --- | --- |
|  | *n = 98* | % |
| Sex |  |  |
| Male | 10 | 10.3 |
| Female | 87 | 89.7 |
| Diverse |  |  |
| Highest professional qualification |  |  |
| Student | 2 | 2.1 |
| Nurse with HF/ND II | 45 | 46.4 |
| Nurse with bachelor's degree | 27 | 27.8 |
| Nurse with master's degree | 9 | 9.3 |
| Nurse with MAS/HöFa | 13 | 13.4 |
| Different | 1 | 1 |
|  |  |  |
| Demographic characteristic | Mean/ Median | Standard deviation |
| Age (years) | 35.49/31 | 11.96 |
| Years of professional experience | 13.72/15 | 10.97 |
| Time to complete survey (seconds) | 378.95/382 | 103.23 |

*Note.* ^a^ The corresponding valid percent values are indicated. Valid percent values were calculated by excluding unanswered questions from the calculations of the percentages.

**Appendix- Table 4**

*Demographic characteristics short version of survey (without imputation of missing values)*

| Demographic characteristic | No. of participants | Valid percent values^a^ |
| --- | --- | --- |
|  | *n = 150* | % |
| Sex |  |  |
| Male | 17 | 11.4 |
| Female | 131 | 87.9 |
| Diverse | 1 | 0.7 |
| Highest professional qualification |  |  |
| Student | 1 | 0.7 |
| Nurse with HF/ND II | 72 | 48 |
| Nurse with bachelor's degree | 31 | 20.7 |
| Nurse with master's degree | 22 | 14.7 |
| Nurse with MAS/HöFa | 18 | 12 |
| Different | 6 | 4 |
| Hospital |  |  |
| CCCZ | 23 | 23 |
| Treatment Center A | 20 | 20 |
| Treatment Center B | 7 | 7 |
| Treatment Center C | 48 | 48 |
| Undisclosed | 52 | 52 |
|  |  |  |
| Demographic characteristic | Mean/Median | Standard deviation |
| Age (years) | 38.5/ 31 | 12.56 |
| Years of professional experience | 16.9/ 11 | 12.72 |
| Time to complete survey (seconds) | 285.8/ 257.50 | 110.31 |

*Note.* ^a^ The corresponding valid percent values are indicated. Valid percent values were calculated by excluding unanswered questions from the calculations of the percentages.

**Appendix- Table 5**

*Statements long version of survey and distribution of responses*

| Statement | Code | Disagree | Rather not agree | Undecided | Rather agree | Agree |
| --- | --- | --- | --- | --- | --- | --- |
|  |  | % | % | % | % | % |
| Screening with the DT and PL is difficult… |  |  |  |  |  |  |
| Because screening is repeated too frequently. | S102_13 | 14.9 | 25.5 | 19.1 | 31.9 | 8.5 |
| Because patients are too exhausted. | S102_04 | 6.2 | 19.6 | 17.5 | 43.3 | 13.4 |
| Because there is no time for it. | S102_02 | 17.5 | 37.1 | 18.6 | 21.6 | 5.2 |
| Because other topics and deadlines have priority over the DT. | S102_03 | 12.6 | 28.4 | 16.8 | 30.5 | 11.6 |
| Because patients do not have time on the day of admission. | S102_05 | 10.3 | 17.5 | 17.5 | 35.1 | 19.6 |
| Because patients do not understand the DT linguistically (e.g., foreign language). | S102_06 | 3.2 | 17.9 | 16.8 | 45.3 | 16.8 |
| Because patients do not understand the DT cognitively (e.g., in case of delirium). | S102_07 | 8.3 | 34.4 | 17.7 | 29.2 | 10.4 |
| Because patients have difficulties in assessing their distress. | S102_08 | 8.2 | 25.8 | 17.5 | 39.2 | 9.3 |
| Because patients are too poorly informed about the meaning of screening through written explanations. | S102_09 | 11.7 | 28.7 | 19.1 | 30.9 | 9.6 |
| Because patients question its necessity due to the lack of apparent clinical benefit for themselves. | S102_10 | 7.4 | 12.6 | 15.8 | 47.4 | 16.8 |
| Because the burden for relatives is not inquired about. | S102_11 | 17.4 | 26.1 | 25 | 21.7 | 9.8 |
| Because screening should be done by another / specialized professional (e.g., doctors, nursing expert). | S102_12 | 46.8 | 35.1 | 5.3 | 5.3 | 7.3 |
|  |  |  |  |  |  |  |
| The screening with the DT and PL is difficult… |  |  |  |  |  |  |
| Because I question its necessity due to the lack of apparent clinical benefit. | S103_01 | 37.9 | 29.5 | 6.3 | 21.1 | 5.3 |
| Because I cannot support the screening guidelines. | S103_02 | 42.1 | 30.5 | 10.5 | 13.7 | 3.2 |
| Because patients will not answer honestly. | S103_03 | 19.6 | 29.3 | 17.4 | 29.3 | 4.3 |
| Because screening means additional work for me without any additional benefit. | S103_04 | 34.4 | 30.2 | 9.4 | 21.9 | 4.2 |
| Because I had a bad experience with screening. | S103_05 | 58.5 | 23.4 | 9.6 | 7.4 | 1.1 |
| Because in my opinion the presence of relatives influences the answers. | S103_06 | 18.4 | 32.2 | 12.6 | 28.7 | 8 |
| Because certain patient groups must be treated with caution. | S103_07 | 37.5 | 26.1 | 17 | 11.4 | 8 |
| Because I feel I have not received enough training for this. | S103_08 | 68.8 | 21.9 | 7.3 | 1 | 1 |
| Because patients are not referred for psycho-oncological support after all. | S103_10 | 40.7 | 31.4 | 9.3 | 11.6 | 7 |
|  |  |  |  |  |  |  |
| The discussion of DT- and PL-results with patients is difficult… |  |  |  |  |  |  |
| Because there is no private location for conversations. | S104_01 | 24.5 | 22.3 | 6.4 | 30.9 | 16 |
| Because there isn’t enough time. | S104_02 | 8.2 | 20.6 | 15.5 | 41.2 | 14.4 |
| Because I feel, I have not received enough training. | S104_03 | 61.1 | 28.4 | 6.3 | 3.2 | 1.1 |
| Because a relationship of trust between nurses and patients has not yet been established on the day of admission. | S104_04 | 13.5 | 18.8 | 17.7 | 33.3 | 16.7 |
| Because I feel uncomfortable talking about topics such as sexuality. | S104_05 | 46.4 | 25.8 | 13.4 | 11.3 | 3.1 |
| Because I am afraid of not being able to deal with the emotions of patients. | S104_06 | 58.8 | 27.8 | 5.2 | 7.2 | 1 |
| Because I cannot offer appropriate interventions to the patients. | S104_07 | 29.9 | 29.9 | 13.4 | 16.5 | 10.3 |
| Because I am afraid of hurting patients by addressing certain topics. | S104_08 | 43.3 | 39.2 | 8.2 | 7.2 | 2.1 |
| Because I am not on the same wavelength the patients. | S104_09 | 37.2 | 31.9 | 14.9 | 13.8 | 2.1 |
| Because patients prefer to talk to other professionals (e.g., oncologists / doctors). | S104_10 | 40.7 | 37.4 | 9.9 | 8.8 | 3.3 |
| Because patients indicate a different level of distress than I subjectively perceive as a nurse. | S104_11 | 27.7 | 23.4 | 24.5 | 23.4 | 1.1 |
| Because topics are not addressed honestly and openly by the patient. | S104_12 | 21.7 | 31.5 | 27.2 | 18.5 | 1.1 |
| Because I am afraid to persuade the patients to do something they do not want to do. | S104_13 | 49 | 26 | 13.5 | 9.4 | 2.1 |
|  |  |  |  |  |  |  |
| Patients are not referred to a specialist service… |  |  |  |  |  |  |
| Because it is not entirely clear what the specialized services can offer. | S105_01 | 34 | 30.9 | 10.6 | 21.3 | 3.2 |
| Because I am afraid to persuade patients to do something they do not want to do. | S105_08 | 50 | 32.6 | 10.9 | 5.4 | 1.1 |
| Because patients refuse to be referred to social support services despite their distress. | S105_07 | 14.7 | 11.6 | 22.1 | 38.9 | 12.6 |
| Because patients refuse to be referred to psycho-oncology despite their distress. | S105_02 | 10.5 | 11.6 | 22.1 | 40 | 15.8 |
| Because the referral to psycho-oncology must be done by physicians. | S105_03 | 23.7 | 14 | 15.1 | 22.6 | 24.7 |
| Because referrals to specialized services seem too expensive for the clinic. | S105_04 | 49.3 | 19.2 | 13.7 | 11 | 6.8 |
| Because social support services do not have enough capacity to see all patients who need it. | S105_06 | 31 | 22.5 | 11.3 | 15.5 | 19.7 |
| Because psycho-oncologists do not have enough capacity to see all patients who need it. | S105_05 | 29.2 | 20.8 | 9.7 | 23.6 | 16.7 |
|  |  |  |  |  |  |  |
| It is difficult to incorporate the screening results into everyday nursing care… |  |  |  |  |  |  |
| Because screening results are difficult to find in a patient's file. | S107_01 | 36.2 | 27.7 | 6.4 | 25.5 | 4.3 |
| Because there is insufficient professional support for acute psychosocial problems. | S107_02 | 22.2 | 31.1 | 12.2 | 27.8 | 6.7 |
| Because I feel, I have not received enough training for carrying out the screening. | S107_03 | 63.9 | 25.8 | 5.2 | 3.1 | 2.1 |
| Because I feel, I have not received enough training to interpret screening results. | S107_06 | 55.7 | 27.8 | 6.2 | 7.2 | 3.1 |
| Because I lack options for action | S107_04 | 24.7 | 25.8 | 17.5 | 21.6 | 10.3 |
| Because there is insufficient interdisciplinary collaboration on psychosocial problems. | S107_05 | 21.5 | 25.8 | 8.6 | 30.1 | 14 |

**Appendix- Table 6**

*Statements short version of survey and distribution of responses*

| Statement | Code | Disagree | Rather not agree | Undecided | Rather agree | Agree |
| --- | --- | --- | --- | --- | --- | --- |
|  |  | % | % | % | % | % |
| Screening with the DT and PL is difficult... |  |  |  |  |  |  |
| Because screening is repeated too frequently. | V105_13 | 23.2 | 36.2 | 12.3 | 19.6 | 8.7 |
| Because patients do not understand the DT cognitively (e.g., in case of delirium). | V105_07 | 10.4 | 32.6 | 13.2 | 28.5 | 15.3 |
| Because patients are too poorly informed about the meaning of screening through written explanations. | V105_09 | 9.9 | 31.9 | 17.7 | 29.1 | 11.3 |
| Because patients question its necessity due to the lack of apparent clinical benefit for themselves. | V105_10 | 5.5 | 19.3 | 13.1 | **44.8** | **17.2** |
| Because the burden for relatives is not inquired about. | V105_11 | 15.8 | 25.9 | 16.5 | 30.9 | 10.8 |
| Because screening should be done by another / specialized professional (e.g., doctors, nursing expert). | V105_12 | 32.1 | 35.8 | 10.9 | 12.4 | 8.8 |
|  |  |  |  |  |  |  |
| The screening with the DT and PL is difficult... |  |  |  |  |  |  |
| Because I question its necessity due to the lack of apparent clinical benefit. | V103_01 | 38.8 | 30.6 | 11.6 | 12.9 | 6.1 |
| Because screening means additional work for me without any additional benefit. | V103_04 | 29.9 | 36.1 | 8.3 | 15.3 | 10.4 |
| Because in my opinion the presence of relatives influences the answers. | V103_06 | 29.9 | 27.7 | 14.6 | 23.4 | 4.4 |
|  |  |  |  |  |  |  |
| The discussion of DT- and PL-results with patients is difficult… |  |  |  |  |  |  |
| Because there is no private location for conversations. | V101_01 | 17.4 | 21.5 | 12.5 | 31.9 | 16.7 |
| Because there isn’t enough time. | V101_02 | 11 | 22.6 | 8.9 | **42.5** | **15.1** |
| Because a relationship of trust between nurses and patients has not yet been established on the day of admission. | V101_04 | 14.6 | 22.9 | 13.9 | **31.9** | **16.7** |
| Because I feel uncomfortable talking about topics such as sexuality. | V101_05 | 26.7 | 20.5 | 6.8 | 26.7 | 19.2 |
| Because I am afraid of not being able to deal with the emotions of patients. | V101_06 | 27.7 | 16.2 | 8.1 | 25 | 23 |
|  |  |  |  |  |  |  |
| Patients are not referred to a specialist service… |  |  |  |  |  |  |
| Because it is not entirely clear what the specialized services can offer. | V102_01 | 24.5 | 23.7 | 13.7 | 20.1 | 18 |
| Because psycho-oncologists do not have enough capacity to see all patients who need it. | V102_05 | 30.9 | 34.6 | 9.6 | 16.9 | 8.1 |
|  |  |  |  |  |  |  |
| It is difficult to incorporate the screening results into everyday nursing care… |  |  |  |  |  |  |
| Because screening results are difficult to find in a patient's file. | V104_01 | 44.4 | 36.8 | 4.2 | 11.1 | 3.5 |
| Because I lack options for action. | V104_04 | 32.4 | 30.3 | 10.6 | 19.6 | 7 |
| Because there is insufficient interdisciplinary collaboration on psychosocial problems. | V104_05 | 19.6 | 27.5 | 10.9 | 31.2 | 10.9 |

*Note.* Bold type = high agreement in both the initial 53-item version and the 24-item version.

**Appendix - Table 7**

*Loadings on the extracted 6 principal component model.*

| Variables | Dim.1 | Dim.2 | Dim.3 | Dim.4 | Dim.5 | Dim.6 |
| --- | --- | --- | --- | --- | --- | --- |
| SD01 |  |  |  | 5.2 |  | 7.3 |
| SD02_01 |  |  |  | 21.5 |  |  |
| SD20_02 |  |  |  | 20.3 |  | 4.1 |
| SD12 |  |  |  | 6.6 |  |  |
| SC01 |  |  |  | 0.0 |  |  |
| S102_13 |  | 9.5 |  |  |  |  |
| S102_04 |  | 5.6 |  |  | 4.6 | 5.4 |
| S102_02 |  |  |  | 3.5 |  | 3.1 |
| S102_03 | 2.6 |  |  |  |  |  |
| S102_05 |  | 6.8 |  |  |  |  |
| S102_06 |  |  | 4.2 |  |  |  |
| S102_07 |  |  |  | 3.1 |  |  |
| S102_08 |  |  |  |  | 11.0 |  |
| S102_09 |  |  |  | 5.0 |  |  |
| S102_10 |  | 9.2 |  |  | 3.0 |  |
| S102_11 |  |  |  |  | 3.2 | 5.4 |
| S102_12 |  |  |  |  |  | 3.0 |
| S103_01 |  | 9.4 |  |  |  |  |
| S103_02 |  | 3.5 |  |  |  |  |
| S103_03 |  |  |  |  | 10.6 |  |
| S103_04 |  | 6.9 |  |  |  |  |
| S103_05 | 2.9 |  |  |  |  |  |
| S103_06 | 1.6 |  |  |  |  |  |
| S103_07 |  |  |  |  |  | 6.0 |
| S103_08 |  |  | 8.3 |  |  |  |
| S103_10 |  | 3.1 |  |  |  |  |
| S104_01 |  |  | 5.2 |  | 3.4 |  |
| S104_02 |  |  | 3.8 | 5.3 |  |  |
| S104_03 |  |  | 6.6 |  |  |  |
| S104_04 |  |  |  |  | 2.8 |  |
| S104_05 |  |  |  |  |  | 6.3 |
| S104_06 | 3.5 |  | 4.4 |  |  | 3.5 |
| S104_07 |  |  |  |  | 4.1 | 3.3 |
| S104_08 | 3.4 |  | 5.5 |  |  |  |
| S104_09 |  |  |  |  |  | 5.2 |
| S104_10 |  |  |  |  | 3.5 |  |
| S104_11 |  |  |  |  | 11.1 |  |
| S104_12 |  |  |  |  | 6.6 |  |
| S104_13 |  |  |  |  |  | 3.8 |
| S105_01 | 2.1 |  |  |  |  |  |
| S105_08 |  |  |  |  |  | 5.4 |
| S105_07 |  |  | 2.6 |  |  |  |
| S105_02 |  |  | 3.4 |  |  | 4.2 |
| S105_03 |  | 7.8 | 3.5 |  |  |  |
| S105_04 | 2.4 |  |  |  |  |  |
| S105_06 |  |  | 4.1 | 3.1 |  |  |
| S105_05 |  |  | 5.3 | 3.7 |  |  |
| S107_01 | 2.2 |  |  |  |  |  |
| S107_02 |  |  |  |  | 4.0 |  |
| S107_03 |  |  | 5.4 |  |  |  |
| S107_06 |  |  | 4.4 |  |  |  |
| S107_04 | 3.0 |  |  |  | 7.8 |  |
| S107_05 |  |  | 3.6 |  |  |  |

*Note.* Only loadings that either are the highest of one question or are higher than 3 are presented for clarity. See Appendix - Table 1 for definition of variables (questions). Dim = principal components.

, issues in the relationship between patients and those responsible for screening and

**Appendix - Table 8**

*Confirmatory factor analysis: Final principal component model obtained*

| Principal component | Loadings | SE | z-value | P(>\|z\|) | Std.lv | Std.all |
| --- | --- | --- | --- | --- | --- | --- |
| difficulties in dealing with emotions of patients during screening |  |  |  |  |  |  |
| S104_06 | 1 |  |  |  | 1.439 | 0.918 |
| S105_01 | 0.667 | 0.086 | 7.712 | 0 | 0.959 | 0.658 |
| doubts in the clinical benefits of screening |  |  |  |  |  |  |
| S102_13 | 1 |  |  |  | 0.421 | 0.327 |
| S103_01 | 2.422 | 0.734 | 3.3 | 0.001 | 1.021 | 0.807 |
| S103_04 | 3.022 | 0.929 | 3.252 | 0.001 | 1.274 | 0.929 |
| doubts in the screening procedure |  |  |  |  |  |  |
| S104_01 | 1 |  |  |  | 0.461 | 0.346 |
| S107_05 | 1.518 | 0.432 | 3.519 | 0 | 0.7 | 0.547 |
| socio-economic factors of screeners |  |  |  |  |  |  |
| SD02_01 | 1 |  |  |  | 1.431 | 0.959 |
| SD20_02 | 0.874 | 0.103 | 8.509 | 0 | 1.25 | 0.885 |
| SD12 | 0.401 | 0.112 | 3.59 | 0 | 0.574 | 0.349 |
| issues in the relationship between patients and those responsible for screening |  |  |  |  |  |  |
| S107_04 | 1 |  |  |  | 0.746 | 0.576 |
| S104_04 | 0.999 | 0.197 | 5.071 | 0 | 0.745 | 0.578 |
| lack of training of screeners |  |  |  |  |  |  |
| S102_11 | 1 |  |  |  | 0.148 | 0.117 |
| S104_05 | 5.642 | 3.121 | 1.808 | 0.071 | 0.838 | 0.554 |

*Note.* Principal component = See appendix – table 1 for definition of variables (questions). SE = standard error, P(>|z|) = p-value associated with z-value, Std.lv = standardized latent variables, Std.all = standardized latent and observed variables

**Appendix – Figure 1**

*Scree plot*


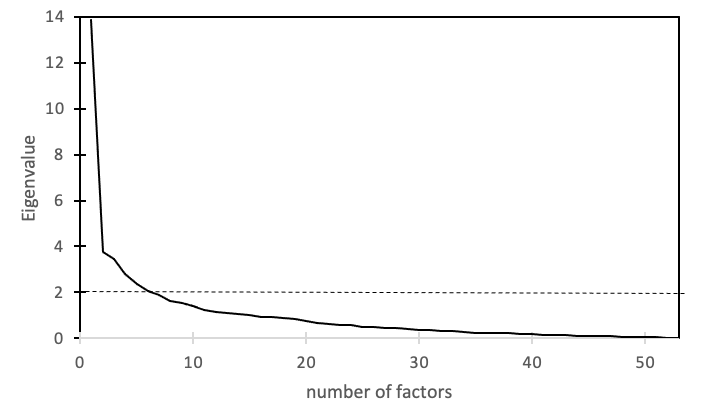


*Note.* Number of factors = number of components.
